# Radiomics in Pituitary Adenomas: A Systematic Review of Clinical Applications and Predictive Models

**DOI:** 10.3390/jcm14186595

**Published:** 2025-09-18

**Authors:** Edoardo Agosti, Marcello Mangili, Pier Paolo Panciani, Lorenzo Ugga, Vittorio Rampinelli, Marco Ravanelli, Alessandro Fiorindi, Marco Maria Fontanella

**Affiliations:** 1 Neurosurgery Unit, Department of Medical and Surgical Specialties, Radiological Sciences and Public Health, University of Brescia, 25123 Brescia, Italy; m.mangili005@studenti.unibs.it (M.M.); pierpaolo.panciani@unibs.it (P.P.P.); alessandro.fiorindi@asst-spedalicivili.it (A.F.); 2 Department of Advanced Medical and Surgical Sciences, University of Campania “Luigi Vanvitelli”, 80131 Naples, Italy; lorenzo.ugga@gmail.com; 3 Unit of Otorhinolaryngology—Head and Neck Surgery, Department of Surgical Specialties, Radiological Sciences, and Public Health, University of Brescia, 25123 Brescia, Italy; vittorio.rampinelli@unibs.it; 4 Radiology Unit, Department of Medical and Surgical Specialties, Radiological Sciences and Public Health, University of Brescia, 25123 Brescia, Italy; marco.ravanelli@unibs.it

**Keywords:** radiomics, machine learning, pituitary adenoma, magnetic resonance imaging, systematic review

## Abstract

**Background**: Radiomics offers quantitative, high-dimensional data from conventional imaging and holds promise for improving diagnosis and treatment of pituitary adenomas (PAs). This systematic review aimed to synthesize current clinical applications of radiomics in PAs, focusing on diagnostic, predictive, and prognostic modeling. **Methods**: This review followed the PRISMA 2020 guidelines. A systematic search was performed in PubMed, Scopus, and Web of Science on 10 January 2024, and updated on 5 March 2024, using predefined keywords and MeSH terms. Studies were included if they evaluated radiomics-based models using MRI for diagnosis, classification, consistency, invasiveness, treatment response, or recurrence in human PA populations. Data extraction included study design, sample size, MRI sequences, feature types, machine learning algorithms, and model performance metrics. Study quality was assessed via the Newcastle-Ottawa Scale. Descriptive statistics summarized study characteristics; no meta-analysis was performed due to heterogeneity. **Results**: Out of 341 identified articles, 49 studies met inclusion criteria, encompassing a total of more than 9350 patients. The majority were retrospective (43 studies, 88%). MRI sequences used included T2-weighted imaging (35 studies, 71%), contrast-enhanced T1WI (34 studies, 69%), and T1WI (21 studies, 43%). PyRadiomics was the most common feature extraction tool (20 studies, 41%). Machine learning was employed in 43 studies (88%), predominantly support vector machines (16 studies, 33%), random forests (9 studies, 18%), and logistic regression (9 studies, 18%). Deep learning methods were applied in 17 studies (35%). Regarding diagnostic performance, 22 studies (45%) reported an (AUC) ≥0.85 in test datasets. External validation was performed in only 6 studies (12%). Radiomics applications included histological subtype prediction (14 studies, 29%), surgical outcome prediction (13 studies, 27%), invasiveness assessment (7 studies, 15%), tumor consistency evaluation (8 studies, 16%), and response to medical or radiotherapy treatments (3 studies, 6%). One study (2%) addressed automated segmentation and volumetry. **Conclusions**: Radiomics enables high-performance, noninvasive prediction of PA subtypes, consistency, invasiveness, treatment response, and recurrence, with 22 studies (45%) reporting AUC ≥0.85. Despite promising results, clinical translation remains limited by methodological heterogeneity, low external validation (6 studies, 12%), and lack of standardization.

## 1. Introduction

Pituitary adenomas (PAs) represent approximately 10–15% of primary intracranial neoplasms [1,2,3]. While commonly benign, they are clinically significant due to hormone hypersecretion syndromes and their potential to exert mass effects on adjacent anatomical structures. This dual mechanism of impact can severely compromise patient quality of life [1]. Importantly, over 30% of PAs demonstrate invasive behavior, infiltrating the cavernous sinuses (CSs), bony structures, the hypothalamus, or the internal carotid artery, which can complicate both diagnosis and treatment planning [4,5].

Magnetic resonance imaging (MRI) is the primary imaging modality used in the diagnostic work-up of PAs. It plays a crucial role not only in the initial identification and anatomical localization of the lesion, but also in long-term monitoring of tumor progression or recurrence. The strengths of MRI lie in its high-resolution, multiplanar capabilities and superior soft tissue contrast. However, the traditional approach to MRI interpretation is qualitative and dependent on radiologist expertise, leading to potential inter-observer variability and missed subtle findings. The subjective nature of this evaluation has spurred the development of more objective, reproducible tools [6].

Radiomics is a computational technique that extracts a vast array of quantitative features from standard medical imaging, offering a high-dimensional analysis of tumor phenotypes that surpass visual assessment. These features include intensity, texture, shape, and spatial relationships, capturing microstructural heterogeneity and tumor biology [7]. The field has expanded significantly in recent years, particularly when integrated with artificial intelligence (AI) and machine learning (ML). These tools can build predictive models that automatically interpret complex radiomic data, enhancing diagnostic accuracy, prognostication, and treatment personalization [8,9,10,11].

Radiomics has already demonstrated utility in various domains relevant to the clinical management of Pas [12]. Studies have shown its potential in differentiating PAs from other sellar and suprasellar lesions, as craniopharyngiomas and Rathke cleft cysts [13,14]. Additionally, radiomics has proven valuable in preoperative histological subtyping [15,16,17,18,19,20,21,22,23,24,25,26,27,28]. For instance, models trained on multiparametric MRI data have accurately distinguished subtypes, such as silent corticotroph adenomas (SCAs) and null cell adenomas (NCAs), entities that may otherwise be clinically silent yet have different prognostic implications [21,22,23,24]. Furthermore, PA consistency, a key factor in predicting the ease and success of surgical resection, has been correlated with specific radiomics features [29,30,31,32,33,34]. Radiomics also aids in evaluating invasiveness, particularly the prediction of CS invasion through features that align with higher Knosp grades [35,36,37,38,39,40,41]. Radiomic models have also shown promise in predicting responsiveness to pharmacologic treatments, like somatostatin receptor ligands in patients with acromegaly, and in forecasting the effects of radiotherapy. These predictions allow for more tailored treatment plans and may help avoid ineffective or unnecessary therapies [42,43]. Lastly, radiomics can contribute to the prognostication of recurrence and progression after surgical resection [44,45,46,47].

Despite the growing body of literature, no recent systematic review has comprehensively summarized the current applications of radiomics for PAs, the models that have been proposed, and which approaches have shown the most promising performance [48]. Moreover, even if several studies have been published in recent years covering a wide range of applications from histological characterization and invasion prediction to response assessment and outcome forecasting, these findings often are still fragmented across the literature. Therefore, the present systematic review aims to collect, organize, and analyze these updated data to provide a clear and accessible summary of the state-of-the-art in radiomics for PAs and its applications.

## 2. Materials and Methods

This systematic review was conducted in accordance with the Preferred Reporting Items for Systematic Reviews and Meta-Analyses (PRISMA) guidelines [49]. Two reviewers (E.A. and M.M.) independently performed a comprehensive search of the PubMed, Scopus, and Web of Science databases. The initial search was conducted on 10 January 2025, with a final update on 5 May 2025. The articles identified through this search were published between 2018 and 2025.

The search strategy utilized a combination of keywords and MeSH terms, including “radiomics,” “artificial intelligence,” “machine learning,” “pituitary adenoma,” “pituitary neuroendocrine tumor,” “PitNET,” “MRI,” and “magnetic resonance imaging.” Boolean operators (AND/OR) were employed to construct the search query as follows: (“radiomics” OR “machine learning” OR “artificial intelligence”) AND (“pituitary adenoma” OR “PitNET” OR “pituitary neuroendocrine tumor”) AND (“MRI” OR “magnetic resonance imaging”). Additional relevant studies were identified by manually screening the reference lists of all selected articles.

Studies were eligible for inclusion if they were published in English, involved human subjects, and investigated the application of radiomics (alone or in combination with AI-based methods) for the diagnosis, classification, prediction, or treatment response assessment of PAs using MRI-based features. Both retrospective and prospective original studies were considered. Articles were excluded if they were review articles, editorials, conference abstracts, purely technical or methodological papers without clinical data, or if they involved only phantom or animal models. Studies lacking clearly reported radiomic methodology, outcome measures, or validation procedures were also excluded.

All references were managed using EndNote X9, and duplicate records were removed prior to screening. Two reviewers (E.A. and M.M.) independently screened titles and abstracts for relevance. Full-text screening was then performed on all articles meeting initial inclusion criteria. Discrepancies at any stage were resolved by discussion or consultation with a third reviewer (P.P.P.).

### 2.1. Data Extraction

Data were extracted systematically from each included article using a pre-defined standardized data collection sheet. Extracted variables included: first author, year of publication, study design, population size, radiomics pipeline (including image acquisition, segmentation, feature extraction, and model development), type of MRI sequences used, clinical endpoint(s) investigated (e.g., differential diagnosis, subtype classification, tumor consistency, invasion, treatment response, or recurrence), and performance metrics (e.g., accuracy, sensitivity, specificity, and area under the curve, i.e., AUC). Additional details were collected on validation methods (internal or external) and the use of AI or ML algorithms.

### 2.2. Outcomes

The primary objective of this review was to synthesize and characterize the current applications of radiomics in the clinical management of PAs, including diagnostic differentiation, subtype classification, prediction of invasiveness or consistency, treatment response forecasting, and recurrence risk stratification. Secondary outcomes included an evaluation of the types and performance of models developed, common methodological patterns, and the overall level of validation achieved across studies.

### 2.3. Radiomics Quality Assessment

A qualitative assessment using the Radiomics Quality Score (RQS) framework was performed with the aim of critically analyzing and comparing the clinical applicability and translational potential of the various radiomic models developed [50]. Evaluation of the included studies was conducted according also to the IBSI compliance checklist to examine their reproducibility and adherence to technical standards [51]. Since many items in the IBSI checklist overlap with those in the RQS checklists, we included only the items relevant to image pre-processing steps (Table 1).

### 2.4. Risk of Bias Assessment

**Figure 1:**
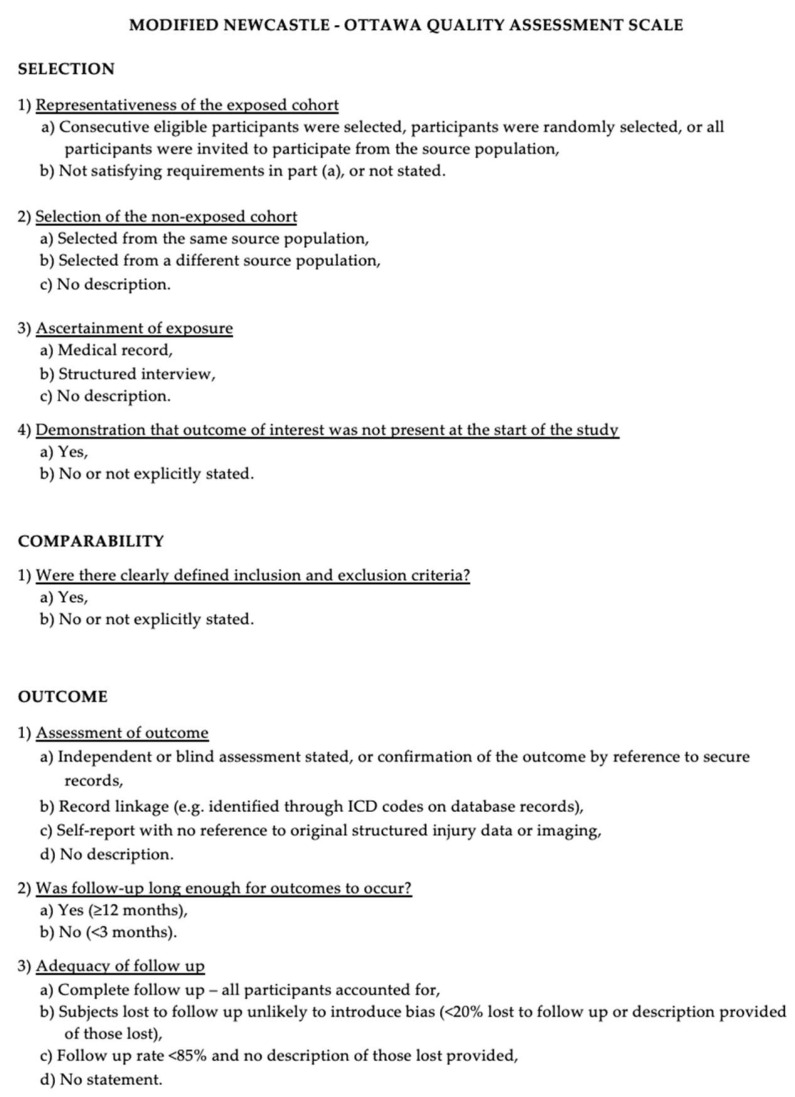
Newcastle-Ottawa Scale variables.

The quality of the observational studies included in the review was evaluated using the Newcastle-Ottawa Scale (NOS), which assesses studies across three domains: selection, comparability, and outcome assessment (Figure 1).

Each study could receive a maximum of 9 points. Those scoring below 7 were classified as low quality and excluded from the analysis. Studies that met or exceeded this threshold were retained, and their quality scores were considered during subgroup analyses and narrative synthesis to explore potential sources of bias. A detailed breakdown of NOS scores for each study is available in Table S1.

### 2.5. Statistical Analysis

Descriptive statistics were used to summarize study characteristics, radiomics applications, and model performance metrics. Results were presented using medians, ranges, and proportions, where appropriate. Due to methodological heterogeneity across studies—including differences in imaging protocols, feature selection methods, and outcome definitions—a formal meta-analysis was not performed. All data processing and visualization were conducted using R statistical software (version 4.2.0) (https://www.r-project.org, accessed on 3 March 2025).

## 3. Results

### 3.1. Literature Review

**Figure 2:**
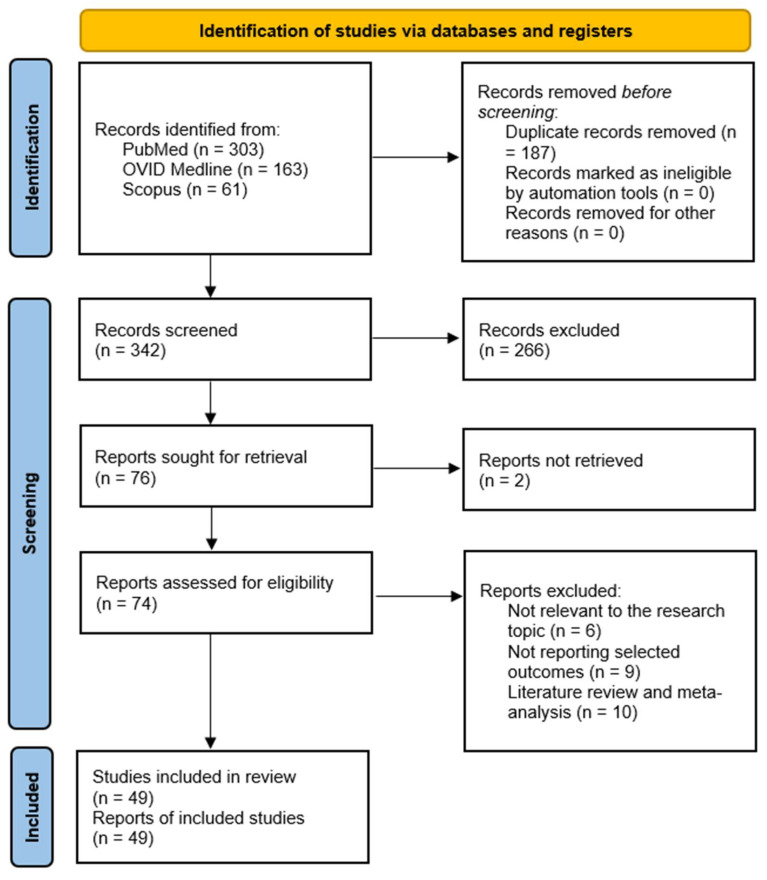
PRISMA flow chart.

After removing duplicates, 340 articles were identified. Following a review of titles and abstracts, 76 studies were selected for full-text screening. Of the 74 articles assessed for eligibility, 49 met the inclusion criteria and were retained for the final analysis. The remaining 25 studies were excluded based on the following reasons: 10 were systematic reviews or meta-analyses, 9 did not report selected outcomes and 6 were unrelated to the research question. The articles included in the final analysis were published between 2018 and 2025. An overview of the selection process is presented in the PRISMA flowchart (Figure 2). PRISMA checklist is available in Table S2.

### 3.2. Data Analysis

A total of 49 studies were included in the systematic review, published between 2018 and 2025, investigating the role of radiomics in the assessment and classification of PAs. The total patient population across all studies was approximately 9351, with individual study sample sizes ranging from 24 to 1045 patients. Of these, 30 studies (61%) reported explicit training and test data splits, with 6 studies (12%) including separate validation cohorts. Regarding MRI sequences, were used T1-weighted imaging (T1WI) in 21 studies (43%), T2-weighted imaging (T2WI) in 35 studies (71%), contrast-enhanced T1WI (CE-T1WI) in 34 studies (69%) and T2-weighted imaging FLAIR (T2WI-FLAIR) in 1 study (2%).

In terms of analytical methodology, shallow ML approaches were utilized in 43 studies (88%), while deep learning (DL) architectures were applied in 17 studies (34%), with some studies using both. The most common ML algorithms were support vector machine (SVM) (19 studies, 44%), random forest (RF) (8 studies, 19%), logistic regression (LR) (8 studies, 19%), and k-nearest neighbors (7 studies, 16%). For DL, convolutional neural networks (CNNs) were used in 8 studies (47%), with architectures such as ResNet, DenseNet, and U-net represented. Feature extraction was performed using PyRadiomics in 23 studies (47%), while Matlab-based custom tools were used in 4 studies (8%), and other platforms including 3D Slicer or proprietary scripts in the remaining cases.

The types of radiomic features extracted varied, but first-order statistics (FOS) were used in 18 studies (37%), shape-based features in 20 studies (41%), and texture features (e.g., GLCM, GLRLM, GLSZM, NGTDM, GLDM) in 25 studies (51%). Wavelet-transformed features were included in 10 studies (20%), and higher-order features (e.g., histogram-based or delta-radiomics) were reported in 5 studies (10%).

Regarding tumor subtype, 35 studies (72%) included PAs with any hormonal secretion profile, 7 studies (14%) focused on functional PAs, including GH-secreting, ACTH, TSH, FSH/LH, and prolactinomas, while other 7 (14%) focused on non-functioning PAs (NFPA). The diagnostic performance was reported using AUC or accuracy values in all studies, with AUCs ranging from 0.55 to 1.00. Most models demonstrated high performance, with 22 studies (45%) reporting AUC values ≥0.85 in the test set. Further-more, 6 studies (12%) included external validation, strengthening the generalizability of their findings (Table 2).

The most frequently represented field was the prediction of histopathological features, explored in 14 studies (29%), making it the dominant area of investigation. Within this category, the preoperative prediction of Ki-67 index was the most common specific endpoint, analyzed in 5 studies, followed by efforts to distinguish subtypes such as SCAs, NCAs, and Tpit/Pit-1/SF-1 families, and to classify functional versus non-functional adenomas. The second most common field was the prediction of response to surgical treatment, addressed in 13 studies (27%). Of these, post-surgical recurrence or regrowth was the most studied endpoint, featured in 4 studies, indicating its clinical relevance. This was followed by investigations into visual outcome, biochemical remission, and intraoperative cerebrospinal fluid leak prediction. The prediction of tumor consistency and prediction of invasiveness were respectively addressed in 8 and 7 studies (16% and 14%). For consistency, all studies focused on distinguishing soft from fibrous tumors, while the invasiveness category primarily targeted CS invasion (6 studies), and to a lesser extent suprasellar extension. Studies on diagnosis of PAs accounted for 4 publications (8%), with most evaluating automated tumor detection from brain MRI, and one study distinguishing cystic PAs from Rathke’s cleft cysts. Lastly, prediction of response to non-surgical treatment, including somatostatin analogs and dopamine agonists (DAs), was investigated in 2 studies (4%), and automated tumor segmentation and volumetry was explored in 1 study (2%) (Table 3).

## 4. Discussion

Radiomics has increasingly established itself as a transformative tool in the neuro-oncological field, enabling the extraction of high-dimensional, quantitative features from conventional imaging modalities. In the context of PAs, its applications have rapidly expanded across multiple domains, from diagnostic differentiation and tumor subtyping to surgical outcome prediction and response assessment. This systematic review analyzed 49 studies, encompassing over 9300 patients. It highlights both the promise and limitations of current radiomics research in this field and offers a framework for future directions.

### 4.1. Radiomics Quality Assessment

Through RQS evaluation we observed patterns that echo those identified by Kocak et al. [67] in their recent meta-analysis of 1574 radiomics studies across radiological subspecialties. While the number of published works is steadily increasing, the median RQS remains modest (approximately 30%) reflecting persistent gaps in reproducibility, standardization, and clinical readiness.

Some methodological strengths were recurrently observed. Most studies adequately reported imaging protocols, frequently including acquisition parameters such as magnetic field strength, slice thickness, and TR/TE values. Feature reduction strategies to mitigate overfitting were commonly employed, and performance metrics (particularly ROC curves and AUC values) were almost universally reported, establishing a baseline level of analytical rigor. More recently, an encouraging trend has emerged toward multicenter cohorts and external validation, in contrast to earlier works that relied heavily on single-center data and internal validation techniques.

Nonetheless, several critical limitations persist. None of studies included phantom experiments or test–retest imaging, both of which are essential for evaluating feature robustness across scanners and timepoints. Calibration statistics, integral for assessing the clinical reliability of predictive models, were seldom reported. The integration of radiomic features with non-imaging variables, such as clinical, histopathological, or molecular data, was inconsistent, thus limiting the development of comprehensive, biologically informed models. Most notably, very few studies provided access to code, segmentation masks, or extracted feature sets, undermining transparency and reproducibility, issues explicitly highlighted by the IBSI and by Lambin et al. in their foundational framework [50,51].

Validation remains the principal barrier to clinical applicability. Over 90% of the studies reviewed relied exclusively on internal validation methods, with true external validation still rarely implemented. This raises substantial concerns regarding the generalizability of radiomic signatures across centers, scanners, and patient populations. As Kocak et al. [67] demonstrated, studies employing higher-quality validation strategies consistently achieved superior RQS scores and more reliable results.

This systematic analysis highlights also a marked heterogeneity in adherence to the IBSI preprocessing guidelines, which are crucial for ensuring reproducibility, comparability of extracted features and for enabling clinical translation. Overall, a bimodal distribution emerges: on one side, radiomics-oriented studies, often published in the last five years, present well-defined and transparent pipelines that closely follow IBSI standards; on the other, many deep learning–based works reduce preprocessing to minimal or non-standardized steps. High-quality studies (adherence to checklist >70%), typically report isotropic voxel resampling with explicit target dimensions and sometimes interpolation methods, apply rigorous intensity normalization such as z-score, min–max scaling or sigma-based filtering, include multiscale filters like Laplacian of Gaussian and wavelet with specified parameters, and describe gray-level discretization either through fixed bin width or fixed bin number. They also provide robust ROI processing, often with manual or semi-automatic three-dimensional segmentation validated by interobserver reproducibility measures such as ICC or Dice, and in several cases report artifact correction methods like N4ITK bias field correction. These characteristics are consistently observed in reference studies such as those by Kocak et al. [42], Zeynalova et al. [29], Machado et al. [44], Wan et al. [32], Park et al. [26], Zhang et al. [65], Liu et al. [18], Taslicay et al. [13], Shen et al. [47] Cuocolo et al. [30] and Ugga et al. [15], which stand out for their methodological rigor and reproducibility, allowing replication and multicentric integration. In contrast, low-quality studies in this terms (adherence to the IBSI checklist <30%) omit voxel isotropic resampling and rely on simple resizing or cropping, neglect intensity normalization, do not apply IBSI-compliant filters, and fail to report discretization of gray levels. Segmentation is often absent or replaced with fixed bounding boxes, and no artifact correction is performed, despite the well-known susceptibility of MRI to inhomogeneities. These deficiencies, seen in works such as those by Staartjes et al. [52], Qian et al. [55], Fang et al. [36], Feng et al. [41], Villalonga et al. [58], Liu et al. [27], Wang et al. [33], and Zhang et al. [61], reflect a tendency to prioritize raw CNN learning over methodological transparency, with the consequence that performance metrics obtained in single-center settings cannot be readily generalized. It should be acknowledged that the IBSI compliance checklist, while essential for assessing reproducibility and methodological rigor in traditional handcrafted radiomics, presents inherent limitations when applied to studies employing deep learning techniques, in particular to CNNs. Many IBSI items are specifically designed to evaluate predefined feature extraction and discretization steps, which are not explicitly performed in CNN-based pipelines. As a result, deep learning studies may receive lower IBSI scores, not necessarily due to inferior quality, but because of a methodological mismatch.

### 4.2. Diagnostic and Subtype Classification

Although the biochemical diagnosis of functioning PAs is relatively straightforward, imaging-based classification remains relevant for differential diagnosis, particularly in the case of silent subtypes or incidentalomas. Numerous studies have explored the use of radiomics in identifying hormonal secretion profiles and tumor subtypes. Baysal et al. [28] demonstrated that artificial neural networks trained on T2WI radiomic features could distinguish seven different hormone-secreting profiles, with AUCs ranging from 0.74 to 0.96, including high performance in distinguishing GH-secreting adenomas (AUC = 0.89) and prolactinomas (AUC = 0.95). Similar subtype classification efforts were conducted by Peng et al. [20], who developed machine learning classifiers (SVM, KNN, NB) capable of distinguishing transcription factor–based PitNET families (Tpit, Pit-1, SF-1) with an AUC up to 0.95 based on multiparametric MRI-derived features.

Specific studies targeted NFPAs, with a focus on histological subtypes like silent corticotroph adenomas (SCAs). Rui et al. [22] and Wang et al. [23] both reported high-performing models for preoperative SCA prediction (AUCs > 0.90), combining radiomic features from T1WI, T2WI, and CE-T1WI sequences with clinical data. Zhang et al. [21] extended this approach to distinguish NCAs, achieving concordance indices up to 0.86 using radiomic nomograms based on T1WI features. Furthermore, in GH-secreting tumors, Park et al. [26] applied radiomics to assess granulation patterns, yielding AUC of 0.83, underscoring radiomics relevance in treatment stratification for acromegaly. A similar model is presented by Liu et al. [27] achieving an AUC of 0.92, with DCA showing that their prediction model had a better net benefit than either the treatment or no treatment schemes when the threshold probability was 0.254 to 0.798.

### 4.3. Prediction of Tumor Consistency

Preoperative assessment of tumor consistency is vital for surgical planning, as fibrous tumors often pose greater technical challenges during resection. Radiomics has demonstrated utility in this domain, primarily through the analysis of T2WI signal textures. Zeynalova et al. [29] used artificial neural networks to predict tumor consistency with an AUC of 0.71, outperforming conventional signal intensity ratios. Cuocolo et al. [30] further refined this approach using an ensemble learning classifier trained on T2WI-derived features, achieving an AUC of 0.99 and an accuracy of 93%.

Other studies expanded this paradigm to include multiparametric MRI. Wan et al. [32] analyzed T1WI, T2WI, and CE-T1WI sequences using 3D segmentation and showed that texture and wavelet features outperformed shape features in consistency prediction (AUC = 0.90). In GH-secreting adenomas, Fan et al. [54] developed an elastic net-based radiomic model with external validation that reached AUCs of 0.83–0.81 when combined with Knosp grade with DCA confirming its net benefit for preoperative surgical planning. Moreover, they validated the constructed MR radiomics model through a completely independent multicenter prospective validation set, offering robustness among different image acquisition protocols. These findings suggest that tumor consistency, a previously subjective and intraoperative assessment, can now be inferred with reasonable accuracy using non-invasive, preoperative imaging and radiomics.

### 4.4. Assessment of Invasiveness and Aggressiveness

Cavernous sinus invasion, as graded by the Knosp classification, significantly impacts surgical decision-making. Radiomics provides an opportunity to enhance preoperative prediction of invasiveness, especially in borderline Knosp grades. Niu et al. [35] developed a radiomic-clinical nomogram that integrated CE-T1WI features such as tumor sphericity and internal carotid artery wrapping, yielding an AUC of 0.90. Their DCA demonstrated that at threshold probabilities above 20%, the radiomics nomogram provided greater net benefit compared to conventional clinical-radiological models or treating all/none strategies. Similarly, Zhang et al. [14] analyzed 3D CE-T1WI radiomic features and reported AUCs of 0.86 (training) and 0.73 (validation) for predicting invasiveness.

Radiomics has also been applied to define broader histopathological aggressiveness criteria, including proliferation indices such as Ki-67 and p53. Ugga et al. [15] showed that T2WI-based features could predict high Ki-67 expression with an AUC of 0.87 using a KNN classifier. Wang et al. [25] took a multimodal approach, combining radiomic features and Knosp grade to identify aggressive tumors (AUC = 0.94), while Fan et al. [16] applied similar methods in acromegalic patients, achieving AUCs of 0.96 and 0.89 across training and test cohorts and the DCA demonstrated that both a radiomic signature and derived nomogram had tangible utility in acromegaly management Liu et al. [18] introduced a novel application of dynamic contrast-enhanced MRI (DCE-MRI) for texture feature extraction. By analyzing pharmacokinetic maps such as Ktrans and Kep, they developed a model that achieved an AUC of 0.96 in distinguishing aggressive from non-aggressive PitNETs. Their DLR model DCA curves demonstrated a strong agreement between predicted and observed outcomes in the internal test set and showed superior clinical benefit in the preoperative prediction of concurrent high Ki-67 expression and PIT-1 positivity, marking an innovative step in functional radiomics.

### 4.5. Prediction of Surgical Outcomes

A key clinical challenge in PA management is the recurrence or progression of residual tumor post-surgery. Galm et al. [68] showed that preoperative T1WI texture metrics such as pixel intensity could stratify recurrence risk in NFPAs, with lower intensity associated with higher recurrence. Machado et al. [44] reported that KNN and RF classifiers trained on CE-T1WI radiomic features achieved AUCs up to 0.98 and 0.96 for recurrence prediction, with 3D segmentation outperforming 2D approaches. Zhang et al. [45] developed a radiomic score using SVMs and demonstrated its value as an independent prognostic factor (AUC = 0.87). In a broader cohort including all adenoma types, Zhang et al. [60] later compared clinical-pathological and radiomic classifiers, reporting improved predictive accuracy with the inclusion of radiomic features (AUC = 0.84 vs. 0.65); moreover DCA results showed that both their delta-radiomic and combined models achieved superior net benefit compared to clinical variables alone, in both development and independent test cohorts.

Post-surgical hormonal remission has also been evaluated. Fan et al. [53] analyzed over 13,000 CE-T1WI features and built a model with AUCs of 0.83–0.81 across training and test cohorts, significantly outperforming models based on clinical parameters alone. They performed a DCA, revealing clinically meaningful benefit at threshold probabilities >13% in the primary cohort and >25% in the validation cohort. These findings indicate that radiomics can contribute meaningfully to post-operative outcome prediction.

### 4.6. Prediction of Response to Medical and Radiotherapy

Radiomics is increasingly being evaluated as a tool to predict the efficacy of pharmacologic therapies, particularly in functioning PAs. Kocak et al. [42] applied texture analysis to T2WIs in acromegaly patients and found that radiomic classifiers (KNN) outperformed qualitative and semi-quantitative signal assessments in predicting response to somatostatin receptor ligands (AUC = 0.85). In this study there were significant differences, with the best performances shown by the quantitative texture analysis based radiomic model, in terms of sensitivity, specificity and AUC-ROC. The other radiomic models were the 2D ROI-based quantitative rSI evaluation model and the 3D segmentation-based quantitative rSI evaluation model, that showed questionable performances if compared to qualitative (visual) rSI evaluation and granulation pattern-based evaluation. Galm et al. [68] correlated T1WI texture parameters with IGF-1 normalization, showing an odds ratio of 5.96 for higher pixel intensity after adjusting for clinical covariates.

Park et al. [43] applied ensemble classifiers to predict DAs response in prolactinoma patients, achieving an AUC of 0.81. Importantly, conventional imaging markers such as T2 intensity or cystic changes were not predictive, highlighting the incremental value of radiomics. Fan et al. [69] further extended the application to radiotherapy outcomes, showing that CE-T1WI-based radiomic models (AUC = 0.92) surpassed clinical-only models (AUC = 0.86). The combined model incorporating both radiomic and clinical data achieved an AUC of 0.96, offering a compelling argument for radiomics-guided treatment stratification in post-operative settings.

### 4.7. Technical and Methodological Considerations

Most of the reviewed studies utilized T1WI, T2WI, and CE-T1WI sequences, with PyRadiomics as the dominant feature extraction tool. Feature types commonly included FOS, shape descriptors, and texture metrics (GLCM, GLRLM, GLSZM), with increasing use of wavelet and higher-order features. Most studies relied on SVMs, RF, and logistic regression models, though DL architectures (e.g., CNNs, ResNet, DenseNet) have become more prevalent in recent years, particularly in larger datasets such as those reported by Zhang et al., Liu et al., and Gargya et al.

Despite high reported accuracies (AUC ≥ 0.80 in 81,6% of studies), generalizability remains a concern. Only 6 studies (12%) performed external validation. Moreover, adherence to radiomics quality standards (e.g., IBSI, RQS) was inconsistently reported, and segmentation protocols varied widely. Few studies applied robustness testing or accounted for variability in imaging acquisition.

### 4.8. Limitations and Future Directions

While current evidence underscores the potential of radiomics to enhance the clinical management of PAs, several limitations hinder its translation into routine practice. Most studies were retrospective and single-center, with substantial heterogeneity in imaging protocols, segmentation methods, feature extraction, and model validation. Only studies by Fan et al. [54] and Li et al. [24] employed external validation, and adherence to radiomics quality standards was inconsistently reported. The absence of standardized pipelines and variability in performance metrics limit reproducibility and comparability. Future research should emphasize prospective, multicenter studies, standardized methodologies, and clinical integration to advance radiomics toward reliable, personalized care in PA management. High-quality research embodies the potential for integration, harmonization, and reproducibility, whereas lower-quality studies, despite innovations in deep learning, often lack the methodological rigor required for reliable translation. The future of the field lies in the convergence of deep learning approaches with IBSI-standardized preprocessing and adherence to best practices outlined in the RQS framework, as reinforced by Kocak et al. [67] and Lambin et al. [50]

## 5. Conclusions

This systematic review highlights the expanding role of radiomics in the clinical management of PAs, demonstrating high diagnostic performance across applications such as subtype classification, tumor consistency, invasiveness, and treatment outcome prediction. Radiomic models achieved AUC ≥0.85 in 45% of studies, primarily using ML and multiparametric MRI. However, clinical adoption remains limited due to methodological heterogeneity, lack of standardized workflows, and scarce external validation (4%). To enable broader translation into the clinical decision-making process, several concrete recommendations should be prioritized: robustness testing through phantom studies, longitudinal imaging, and inter-observer segmentation variability; rigorous validation, with preference for external and multicenter datasets; transparency through the sharing of code, annotations, and feature sets; integrative modeling that combines radiomic, clinical, and molecular data; and an emphasis on clinical applicability, including the use of calibration and decision curve analyses, as well as the design and registration of prospective studies to evaluate radiomic signatures in real-world workflows.

## Figures and Tables

**Table 1:** Radiomics quality assessment through RQS and IBSI checklists.

Author, Year	Method	RQS (36)	IBSI (%)
Zhang, 2018 [21]	Handcrafted	14	43
Niu, 2018 [35]	Handcrafted	16	43
Kocak, 2018 [42]	Handcrafted	12	86
Staartjes, 2018 [52]	Deep Learning	13	14
Zeynalova, 2019 [29]	Handcrafted	14	86
Ugga, 2019 [15]	Handcrafted	14	71
Fan, 2019 [53]	Handcrafted	16	43
Fan, 2019 [16]	Handcrafted	18	43
Fan, 2019 [54]	Handcrafted	17	43
Qian, 2020 [55]	Deep Learning	10	29
Peng, 2020 [20]	Handcrafted	12	43
Cuocolo, 2020 [30]	Handcrafted	14	71
Machado, 2020 [44]	Handcrafted	10	86
Park, 2020 [26]	Handcrafted	13	100
Zhu, 2020 [31]	Deep Learning	9	29
Chen, 2020 [34]	Handcrafted	14	43
Zhang, 2020 [45]	Handcrafted	11	4
Li, 2021 [24]	Deep Learning	13	71
Liu, 2021 [27]	Handcrafted	14	14
Park, 2021 [43]	Handcrafted	12	71
Wan, 2021 [32]	Handcrafted	12	100
Wang, 2021 [33]	Handcrafted	13	14
Zhang, 2021 [56]	Handcrafted	14	14
Zhang, 2021 [57]	Handcrafted	12	43
Baysal, 2022 [28]	Deep Learning	18	57
Chen, 2022 [46]	Multimodal	13	43
Fang, 2022 [36]	Deep Learning	14	29
Feng, 2022 [41]	Deep Learning	15	29
Kim, 2022 [37]	Deep Learning	14	57
Park, 2022 [38]	Deep Learning	13	57
Rui, 2022 [22]	Handcrafted	17	29
Shu, 2022 [17]	Deep Learning	13	57
Villalonga, 2022 [58]	Handcrafted	13	14
Zhang, 2022 [39]	Handcrafted	14	71
Gargya, 2023 [59]	Deep Learning	10	57
Shen, 2023 [47]	Handcrafted	16	86
Wang, 2023 [25]	Handcrafted	16	57
Wang, 2023 [23]	Handcrafted	15	86
Zhang, 2023 [60]	Handcrafted	16	86
Zhang, 2023 [61]	Deeep Learning	17	14
A, 2024 [19]	Handcrafted	14	57
Behzadi, 2024 [62]	Deep Learning	15	43
Da Mutten, 2024 [63]	Deep Learning	13	43
Fang, 2024 [40]	Deep Learning	14	29
Ishimoto, 2024 [64]	Deep Learning	−2	71
Liu, 2024 [18]	Combined	19	86
Taslicay, 2024 [13]	Handcrafted	14	86
Zhang, 2024 [65]	Handcrafted	15	100
Agosti, 2025 [66]	Handcrafted	15	71

**Table 2:** Summary of anamnestic and radiomics data of the studies included in the sys-tematic review. Abbreviations: NF, non-functioning; OC, optic chiasma; ICA, internal ca-rotid artery; GH, growth hormone; IFPA, invasive functional pituitary adenoma; PMA, pi-tuitary macro-adenoma; PRL, prolactin; ACTH, adrenocorticotropic hormone; CPA, cystic pituitary adenoma; RBF-SVM, support vector ma-chine with the radial basis function ker-nel; SVM, support vector machine; MLP, multi-layer perceptron; ANN, artificial neural network; CRNN, convolutional recurrent neural network; kNN, k-nearest neighbors; NB, Naïve Bayes; ET, Extra Trees; RF, Random Forest; LightGBM, light gradient boosting ma-chine; DT, Decision Tree; AdaBoost, Adaptive Boosting; XGBoost, Extreme Gradient Boosting; GBDT, gradient boosting decision tree; BFGS, Broy-den-Fletcher-Goldfarb-Shanno; IF, isolation forest; oSVM, one-class support vector machine; PNN, probabilistic neural network; SNR, signal-to-noise ratio; CNR, con-trast-to-noise ratio; ICA, internal carotid artery; FOS, First Order Statistics; GLCM, Gray Level Gray Level Co-occurrence Matrix; GLRLM, Gray Level Run Length Matrix; GLSZM, Gray Level Size Zone Matrix; NGTDM, Neighboring Gray Tone Difference Matrix; GLDM, Gray Level Dependence Matrix; FOSW, First Order Statistics applied over Wavelet-filtered images; FOSG, First Order Statistics applied over Gradient filtered images; CRNN, Con-volutional Recurrent Neural Network.

Author, Year	Patients				MR Sequences	ML Algorithms Used or AI Model (ML or DL)	Software Used for Features Extraction	Type of Radiomic Features	Tumor Subtype	AUC ROC (or Accuracy %)				
	Total (N)	Training Dataset (%)	Test Dataset (%)	Validation Dataset (%)										
Zhang, 2018 [21]	112	67	33	-	T1WI CE-T1WI	RBF-SVM	MatLab	Intensity Shape and size Texture Wavelet-based	NF	T1WI			Training	0.831
													Test	0.804
										CE-T1WI			Training	0.634
													Test	0.510
Niu, 2018 [35]	194	50	50	-	T2WI CE-T1WI	Linear SVM	MatLab	Intensity Shape and size Texture Wavelet-based ICA wrapped degree	Any				Training	0.852
													Test	0.826
Kocak, 2018 [42]	47	-	-	-	T2WI	kNN C4.5	PyRadiomics (3D-Slicer extension)	FOS GLCM GLRLM GLSZM NGTDM GLDM Wavelet-based	GH	Quantitative TA			0.847	
										ROI-based quantitative rSI			0.581	
Staartjes, 2018 [52]	140	-	-	-	CE-T1WI	MLP (DL) LR	-	-	Any	MLP (DL)			0.962	
										LR			0.86	
Zeynalova, 2019 [29]	55	-	-	-	T2WI	ANN	PyRadiomics	-	Any (PMA)	0.710				
Ugga, 2019 [15]	89	60	40	-	T2WI	kNN	PyRadiomics	Shape FOS GLCM GLRLM GLSZM NGTDM GLDM	Any	0.87				
Fan, 2019 [53]	163	66	34	-	T1WI T2WI CE-T1WI	SVM	Inhouse program written in Matlab 2015b	Intensity Shape and size Texture Wavelet-based	Any (IFPA)	Training			0.832	
										Validation			0.811	
Fan, 2019 [16]	138	65	35	-	T1WI T2WI CE-T1WI	SVM	PyRadiomics	Shape FOS Texture Wavelet-based	GH	Training			0.96	
										Validation			0.89	
Fan, 2019 [54]	188	53	31	16	T1WI T2WI CE-T1WI	SVM LR	PyRadiomics	FOS Shape and size GLCM GLRLM GLSZM Wavelet-based	GH	Training			0.83	
										Validation			0.81	
Qian, 2020 [55]	149	80	20	-	T1WI T2WI	CNN	-	-	Any	-				
Peng, 2020 [20]	235	-	-	-	T1WI T2WI CE-T1WI	SVM kNN NB	PyRadiomics	Shape GLCM GLRLM GLSZM GLDM	Any	SVM		T1WI	0.8762	
												T2WI	0.9549	
												CE-T1WI	0.8806	
										KNN		T1WI	0.8598	
												T2WI	0.9266	
												CE-T1WI	0.7947	
										NB		T1WI	0.8492	
												T2WI	0.9324	
												CE-T1WI	0.8309	
Cuocolo, 2020 [30]	89	80	20	-	T2WI	ET	PyRadiomics	Histogram GLCM GLRLM GLSZM NGTDM GLDM	Any	0.99				
Machado, 2020 [44]	27	-	-	-	CE-T1WI	kNN RF LR SVM MLP	PyRadiomics	FOS GLCM GLRLM GLSZM NGTDM GLDM FOSW FOSG	NF	MLP			0.929	
										RF			0.877	
										SVM			0.860	
										LR			0.929	
										kNN			0.979	
Park, 2020 [26]	69	-	-	-	T2WI	-	PyRadiomics	Shape FOS GLCM GLRLM GLSZM NGTDM GLDM	GH	0.834				
Zhu, 2020 [31]	374	-	-	-	T1WI T2WI	-	DenseNet-ResNet based Autoencoder framework CRNN	-	Any	-				
Chen, 2020 [34]	101	71	29	-	T1WI T2WI CE-T1WI	-	-	-	Any	T1WI		Training	0.90	
												Test	0.91	
										T2WI		Training	0.86	
												Test	0.83	
										CE-T1WI		Training	0.90	
												Test	0.89	
										Combined		Training	0.92	
												Test	0.91	
Zhang, 2020 [45]	50	-	-	-	T2WI CE-T1WI	SVM	Python (v. 3.10.7)	SVR GLCM NGTDM	NF	0.87				
Li, 2021 [24]	185	54	Group 1 24 Group 2 13	9	T1WI T2WI CE-T1WI T2WI-FLAIR	CNN	-	-	Any	Internal Validation 1			0.8063	
										Internal validation 2			0.7881	
										External independent testing			0.8478	
Liu, 2021 [27]	49	-	-	-	T1WI T2WI CE-T1WI	-	PyRadiomics	Shape FOS GLCM GLRLM GLSZM NGTDM GLDM	GH	ROI1		T1C	0.893	
												T1WI	0.918	
												T2WI	0.823	
												Radiomics	0.908	
										ROI2		T1C	0.860	
												T1WI	0.898	
												T2WI	0.812	
												Radiomics	0.880	
Park, 2021 [43]	177	80	20	-	T2WI	RF LightGBM ET	PyRadiomics	FOS GLCM GLRLM GLSZM	PRL	Training			0.81	
										Test			0.81	
Wan, 2021 [32]	156	69	31	-	T1WI T2WI CE-T1WI	RF SVM	MatLab	-	Any (PMA)	0.90				
Wang, 2021 [33]	163	80	20	-	T1WI T2WI CE-T1WI	Linear SVM RF ET kNN DT GDBT AdaBoost MLP XGBoost	PyRadiomics	Knosp grade adenoma volume adenoma diameters OC height ICA contact degree	Any	Linear SVC			0.762	
										RF			0.824	
										ET			0.865	
										KNN			0.920	
										DT			0.597	
										GBDT			0.807	
										AdaBoost			0.817	
										MLP			0.856	
										XGBoost			0.826	
Zhang, 2021 [56]	1045	80	20	-	T1WI T2WI CE-T1WI	GBDT RF AdaBoost XGBoost LR NB DT MLP	-	-	ACTH	XGBoost			0.712	
										GBDT			0.734	
										RF			0.726	
										AdaBoost			0.699	
										NB			0.681	
										LR			0.701	
										DT			0.664	
										MLP			0.700	
										Stacking			0.743	
Zhang, 2021 [57]	131	-	-	-	T2WI	SVM RF LDA	PyRadiomics	Shape FOS GLCM GLRLM GLSZM NGTDM GLDM	Any	SVM			0.824	
										LDA			0.801	
										RF			0.751	
Baysal, 2022 [28]	130	70	15	15	T2WI	ANN (BFGS algorithm)	PyRadiomics	Shape FOS High order features	Any	NF			0.87	
										GH			0.89	
										PRL			0.95	
										ACTH			0.94	
										PH			0.74	
										FSH-LH			0.96	
										TSH			0.95	
Chen, 2022 [46]	78	80	-	20	T2WI CE-T1WI	MLP CNN	-	-	NF	CNN			0.84	
										MLP			0.73	
										Multimodal CNN-MLP			0.85	
Fang, 2022 [36]	371	-	-	-	CE-T1WI	CNN	-	-		Validation fold 1			0.89	
										Validation fold 2			0.98	
										Validation fold 3			0.89	
										Validation fold 4			0.96	
										Validation fold 5			0.93	
Feng, 2022 [41]	695	-	-	-	CE-T1WI	CNN	-		Any	0.98				
Kim, 2022 [37]	67	-	-	-	CE-T1WI	DL	-	Depth of Invasion Degree of contact with intracavernous ICA	Any	1-mm-slice MR			0.79	
										3-mm-slice MR			0.61	
Park, 2022 [38]	104	-	-	-	CE-T1WI	DL	-	-	Any	Reader 1			1-mm-slice MR 0.91 3-mm-slice MR 0.88	
										Reader 2			1-mm-slice MR 0.92 3-mm-slice MR 0.87	
Rui, 2022 [22]	302	80	20	-	T1WI T2WI CE-T1WI	LDA SVM RF GBM	PyRadiomics (3D-Slicer extension)	Shape (3D) Shape (2D) FOS GLCM GLRLM GLSZM NGTDM GLDM	NF	Ensemble			0.927	
Shu, 2022 [17]	261	80	20	-	T2WI CE-T1WI	DL	U-net neural network	-	Any	CE-T1WI			87.4%,	
										T2WI			89.4%	
										CE-T1WI + T2WI			89.2%	
Villalonga, 2022 [58]	144	80	20	-	T1WI T2WI CE-T1WI	IF local outlier factor oSVM	Python	-	Any	-				
Zhang, 2022 [39]	196	90	-	10	CE-T1WI	SVM	PyRadiomics	FOS Shape (3D) GLCM GLSZM GLRLM NGTDM GLDM	Any				0.86	
Gargya, 2023 [59]	-	-	-	-	-	CNN (VGG 16, VGG19, ResNet-50, Inception V3) SVM kNN PNN	-	-	Any	VGG16			89%	
										VGG19			91.5%	
										Resnet 50			91%	
										Inception V3			96%	
Shen, 2023 [47]	114	70	30	-	T1WI T2WI CE-T1WI	LR	R software	Shape FOS GLCM GLRLM GLSZM NGTDM Wavelet-based	NF	Clinical + radiomics features			0.929	
										Only radiomics features			0.844	
Wang, 2023 [25]	246	78	22	-	CE-T1WI	LR	LIFEx	SHAPE_Volume (mL) SHAPE_Volume (vx) SHAPE_Sphericity SHAPE_Surface area SHAPE_Compacity DISCRETIZED_Q3 DISCRETIZED_Kurtosis GLCM GLRM NGLDM GLZLM	Any	Training			0.916	
										Test			0.935	
Wang, 2023 [23]	295	88	12	-	T1WI T2WI CE-T1WI	Elasticnet LinearSVC RF ET kNN DT GBDT AdaBoost MLP XGBoost	PyRadiomics	-	NF	LinearSVC			Training	0.931
													Test	0.937
										Elasticnet			Training	0.908
													Test	0.915
										RF			Training	0.848
													Test	0.82
										ET			Training	0.831
													Test	0.845
										KNN			Training	0.836
													Test	0.762
										DT			Training	0.615
													Test	0.622
										GBDT			Training	0.862
													Test	0.819
										AdaBoost			Training	0.667
													Test	0.793
										MLP			Training	0.903
													Test	0.905
										XGBoost			Training	0.879
													Test	0.868
Zhang, 2023 [60]	130	70	30	-	T2WI	Linear SVM	PyRadiomics	Shape Histogram Texture Wavelet-based	Any	Delta-radiomic model			Training	0.821
													Test	0.811
										Combined model			Training	0.841
													Test	0.840
Zhang, 2023 [61]	220	80	20	-	T2WI	CNN	-	-	Any	-				
A, 2024 [19]	222	67	33	-	T1WI T2WI CE-T1WI	SVM LR RF MLP	PyRadiomics	Shape features FOS GLCM GLRLM GLSZM	Any	Multi-sequence (LR)			Training	0.935
													Test	0.886
													Validation	0.840
									Any	Multi-sequence (MLP)			Training	0.957
													Test	0.913
													Validation	0.758
Behzadi, 2024 [62]	220	70	30	-	T1WI T2WI	CNN	-	-	Any				0.898	
Da Mutten, 2024 [63]	213	91	9	-	CE-T1WI	CNN	-	-	Any	-				
Fang, 2024 [40]	729	89	11	-	CE-T1WI	CNN (ResNet-50)	-	-	Any				0.92	
Ishimoto, 2024 [64]	24	-	-	-	CE-T1WI	DL	-	SNR CNR	Any	-				
Liu, 2024 [18]	247	80	20	-	T1WI T2WI CE-T1WI	LR SVM MLP	PyRadiomics (ML model) ResNet50 (DL model)	FOS Shape Texture	Any	ML model	LR	Training	0.789	
												Test	0.547	
											SVM	Training	0.904	
												Test	0.645	
											MLP	Training	0.812	
												Test	0.620	
										DL model	LR	Training	1.000	
												Test	0.808	
											SVM	Training	1.000	
												Test	0.812	
											MLP	Training	1.000	
												Test	0.765	
										DLR model	LR	Training	1.000	
												Test	0.810	
											SVM	Training	1.000	
												Test	0.810	
											MLP	Training	0.994	
												Test	0.778	
Taslicay, 2024 [13]	65	-	-	-	T1WI T2WI CE-T1WI	SVM LR LGB	PyRadiomics (3D-slicer)	FOS GLCM GLRLM GLSZM NGTDM GLDM	Any (CPA)	SVM			0.956	
										LR			0.956	
										LGB			0.951	
Zhang, 2024 [65]	152	70	30	-	T1WI T2WI CE-T1WI	SVM	PyRadiomics	FOS Shape (3D) Shape (2D) GLCM GLRLM GLSZM NGTDM GLDM	Any	T1WI			Training	0.784
													Test	0.767
										T2WI			Training	0.724
													Test	0.763
										CE-T1WI			Training	0.822
													Test	0.794
										Multiparametric			Training	0.851
													Test	0.847
Agosti, 2025 [66]	394	80	10	10	T2WI	ET	PyRadiomics	Shape FOS GLCM GLRLM GLSZM NGTDM Wavelet-based	Any					0.59

**Table 3:** Summary of radiomics applications for PAs. Abbreviations: CI, confidential interval; CS, cavernous sinus; SF, sellar floor; PIT-1, positive pituitary transcription factor 1; ICA, internal carotid artery; NFPA, non-functioning pituitary adenoma; NCA, null cell adenoma; CSF, cerebrospinal fluid; GTR, gross total resection; PMA, pituitary macroadenoma; SA, somatostatin analogues; DA, dopamine agonist; MRI, magnetic resonance imaging; GH, growth hormone; IFPA, invasive functional pituitary adenoma.

General Field of Application	Specific Endpoint	Author, Year
Prediction of consistency	Distinction between soft and fibrous tumors	Zeynalova, 2019 [29]
		Fan, 2019 [54]
		Cuocolo, 2020 [30]
		Zhu, 2020 [31]
		Chen, 2020 [34]
		Wan, 2021 [32]
		Wang, 2021 [33]
		Agosti, 2025 [66]
Prediction of invasiveness	Prediction of CS invasion	Niu, 2018 [35]
		Fang, 2022 [36]
		Kim, 2022 [37]
		Park, 2022 [38]
		Zhang, 2022 [39]
		Fang, 2024 [40]
	Prediction of SF invasion	Feng, 2022 [41]
Prediction of histopathological features	Preoperative prediction of Ki67	Ugga, 2019 [15]
		Fan, 2019 [16]
		Shu, 2022 [17]
	Prediction of Ki67 and PIT-1	Liu, 2024 [18]
	Distinction between high-risk and low-risk PitNETs (WHO 2021 classifcation)	A, 2024 [19]
	Distinction among Tpit, Pit-1, and SF-1 subfamilies	Peng, 2020 [20]
	Distinction between NCAs and other NFPA subtypes	Zhang, 2018 [21]
	Distinction between SCAs and other NFPA subtypes	Rui, 2022 [22]
		Wang, 2023 [23]
	Distinction between functioning and nonfunctioning PAs	Li, 2021 [24]
	Prediction of aggressiveness (Ki-67 ≥ 3%, positive p53 staining, high mitotic count)	Wang, 2023 [25]
	Prediction of granulation pattern of GH-secreting PAs	Park, 2020 [26]
		Liu, 2021 [27]
	Prediction of hormonal secretion patterns	Baysal, 2022 [28]
Prediction of response to surgical treatment	Prediction of post-surgical recurrence or regrowth	Machado, 2020 [44]
		Zhang, 2020 [45]
		Chen, 2022 [46]
		Shen, 2023 [47]
	Prediction of post-surgical visual outcome	Zhang, 2021 [57]
		Zhang, 2023 [61]
		Zhang, 2023 [60]
		Zhang, 2024 [65]
	Prediction of post-surgical biochemical remission	Fan, 2019 [53]
		Zhang, 2021 [56]
	Prediction of intraoperative CSF leak	Villalonga, 2022 [58]
		Behzadi, 2024 [62]
	Prediction of the likelihood of GTR	Staartjes, 2018 [52]
Prediction of response to non-surgical treatment	Prediction of response to SA in GH-secreting PMAs	Kocak, 2018 [42]
	Prediction of response to DAs	Park, 2021 [43]
Diagnose PAs	Detection of pituitary tumors from brain MRI	Qian, 2020 [55]
		Gargya, 2023 [59]
		Ishimoto, 2024 [64]
	Distinction between pituitary cystic adenomas and Rathke’s cleft cysts	Taslicay, 2024 [13]
Automated tumor segmentation and volumetry	Lesion detection, evaluation of progression of pituitary incidentalomas and detection of residual tumor.	Da Mutten, 2024 [63]

## Data Availability

Data available in a publicly accessible repository.

## Supplementary Materials

The following supporting information can be downloaded at: https://www.mdpi.com/article/10.3390/jcm14186595/s1, Table S1: This table summarized the NOS scores of each study included in the systematic review; Table S2: PRISMA checklist (Reference [70] are cited in the Supplementary Materials).

## Abbreviations

AI = artificial intelligence, AUC = area under the curve, CS = cavernous sinus, CNN = con-volutional neural network, DCE-MRI = dynamic contrast-enhanced magnetic resonance imaging, DL = deep learning, ML = machine learning, FOS = first-order statistics, GLCM = Gray Level Gray Level Co-occurrence Matrix, GLRLM = Gray Level Run Length Matrix, GLSZM = Gray Level Size Zone Matrix, LR = logistic regression, MRI = magnetic resonance imaging, GLDM = Gray Level Dependence Matrix, NCA = null cell adenoma, NFPA = non-functioning pituitary adenoma, NGTDM = Neighboring Gray Tone Difference Matrix, DA = dopamine agonist, PA = pituitary adenoma RF = random forest, SCA = silent cortico-troph adenoma, SVM = support vector machine.
